# IL-7-Treated Periodontal Ligament Cells Regulate Local Immune Homeostasis by Modulating Treg/Th17 Cell Polarization

**DOI:** 10.3389/fmed.2022.754341

**Published:** 2022-02-23

**Authors:** Xin-Yi Yu, Zhao-Qiang Zhang, Jia-Chang Huang, Jia-Yu Lin, Xue-Pei Cai, Chu-Feng Liu

**Affiliations:** ^1^Department of Orthodontics, Stomatological Hospital, Southern Medical University, Guangzhou, China; ^2^Department of Oral and Maxillofacial Surgery, Stomatological Hospital, Southern Medical University, Guangzhou, China

**Keywords:** IL-7, periodontal ligament cells, TGF-β, Treg cells, Notch1 signaling pathway

## Abstract

Both interleukin (IL)-7 and human periodontal ligament cells (hPDLCs) have immunomodulatory properties. However, their combined effect on CD4^+^T cells has never been studied. In this study, we aimed to investigate the effect of conditioned medium of hPDLCs treated with rhIL-7 on the differentiation of CD4^+^T cells into regulatory T cells/T helper 17 cells (Treg/Th17 cells) and observe the effect of IL-7 on the immunomodulatory properties of PDLCs. After hPDLCs were treated with different concentrations of rhIL-7 for 24 h, the collected supernatants were used to incubate CD4^+^T cells for 3 days. A gamma-secretase inhibitor (DAPT) was used to suppress the activation of the Notch1 signaling pathway. Cell proliferation, apoptosis, and necrosis were determined using the cell counting kit-8 (CCK-8) and flow cytometry (FCM). The expressions of forkhead box P3 (Foxp3) in CD4^+^T cells and transforming growth factor (TGF-β) and IL-6 in the supernatants were determined by ELISA. Reverse transcription-quantitative PCR (RT-qPCR), and the Western blot (WB) determined the mRNA levels and protein expression of various target factors. FCM was used to detect the mean fluorescence intensity of PD-L1 in hPDLCs and to analyze the differentiation of Treg/Th17 cells. Our results showed that IL-7 promoted proliferation and inhibited apoptosis in hPDLCs, promoted the expression of TGF-β, PD-L1, Notch1, Jagged1, and Hes1, and inhibited the levels of hypoxia-inducible factor (HIF)-1α and TCF7, whereas the addition of DAPT effectively reversed these effects. Importantly, we found that the conditioned medium of hPDLCs treated with rhIL-7 promoted the polarization of CD4^+^T cells into Treg cells but had no significant effect on the differentiation of Th17 cells. Our study indicated that treatment of PDLCs with IL-7 can promote the polarization of CD4^+^T cells into Treg cells by modulating the expression of inflammatory factors and signaling molecules through activating the Notch1 signaling pathway, thus participating in the regulation of immune homeostasis in the periodontal microenvironment.

## Introduction

The periodontal ligament cells (PDLCs) are the most abundant and powerful cells in the periodontal ligament. They are a heterogeneous cell group comprising fibroblasts, mesenchymal stem cells, and precursor cells, which can differentiate into osteoblasts and odontoblasts (1). These cells are characterized by self-renewal, multidirectional differentiation, and immune regulation and play an important role in regeneration and repair, functional reconstruction, and steady-state maintenance of periodontal tissues (2–4). Fibroblast-like cells in the PDLCs are involved in tissue regeneration and repair through cell proliferation and differentiation into tissue-specific cells and regulation of the immune-inflammatory response. PDLCs play their immunomodulatory role by releasing enzymes and soluble factors and by direct contact between cells, affecting various immune cells and participating in the regulation of immune responses in the local periodontal microenvironment (4, 5). The main key factors mediating these immunomodulatory effects are transforming growth factor (TGF-β), interleukin (IL)-6, and PD-L1 (6). The immunoregulatory factors expressed by PDLCs require activation by CD4^+^T cells to participate in the regulation of periodontal local inflammatory response and osteoclastic activity (7, 8). Therefore, the effect of PDLCs on CD4^+^T cells is the current focus of research (9).

After receiving external signal stimulation, naive CD4^+^T cells can differentiate into different subtypes of functional cells, such as T helper cells Th1, Th2, Th17, or regulatory T (Treg) cells. They also participate in the body's immune response and regulate the regeneration and destruction of bone tissue (10). Th17 cells help in promoting inflammatory responses and bone resorption by secreting pro-inflammatory factors, such as IL-17; Treg cells, as a highly immunosuppressive subgroup of CD4^+^T cells, inhibit the immune response and participate in bone formation by promoting the expression of anti-inflammatory factors, such as TGF-β and IL-10 (11, 12). Treg/Th17 cells antagonize, regulate, and transform through common signaling and metabolic pathways. Therefore, the differentiation and balance of Treg/Th17 cells are important in the occurrence and development of inflammatory responses and to regulate the homeostasis of the local periodontal immune microenvironment and bone metabolism balance (13).

Interleukin-7 is a lymphocyte factor with several immune effects that can regulate the differentiation and survival of CD4^+^T cells and is crucial for regulating T-cell metabolism (14). The IL-7 expression has been found in both periodontitis and orthodontic tooth movement (15, 16). IL-7 directly acting on CD4^+^T cells can induce differentiation into Th1 cells by activating the JAK/STAT5 signaling pathway (17). However, IL-7 has also been shown to regulate autoimmune responses and rebuild and maintain immune tolerance by inducing differentiation and proliferation of Treg cells in mouse models of autoimmune diseases and patients with type 1 diabetes (18, 19). In addition, IL-7 can participate in tissue repair by enhancing the fusion characteristics of rat bone marrow mesenchymal stem cells, promoting their proliferation, differentiation, and migration ability, and inducing the secretion of pro-regeneration factors (20, 21).

However, despite being an important immunomodulatory factor produced by PDLCs during periodontal tissue inflammation, the effects of IL-7 on the immunomodulatory function of PDLCs and their combined effect on CD4^+^T cells are unclear. Therefore, this study aimed to determine the changes in the expression of inflammatory cytokines and signal molecules related to the immune response of hPDLCs after treatment with IL-7 and to investigate their combined effect on the polarization of CD4^+^T cells into Treg/Th17 cells and the possible underlying mechanism.

## Materials and Methods

All protocols were performed after the approval by the Ethical Committee of Southern Medical University and with the informed consent of all volunteers.

### Cell Culture

Extracted teeth needed for the primary culture of hPDLCs were provided by the surgical outpatient department of the Stomatological Hospital of Southern Medical University. Periodontally healthy permanent premolars without root caries that required extraction for orthodontic treatment were obtained from donors aged 16–20 years. Primary hPDLC isolation was performed as described previously (22). The periodontium from the middle one-third of the root surface of each premolar was scraped, and 2 mg/ml collagenase I (Sigma, Germany) was used for its digestion at 37°C for 30 min. Next, periodontium pieces were collected by centrifugation and resuspended with RPMI-1640 (Gibco, USA) containing 10% fetal bovine serum (FBS; Gibco, USA) and 1% penicillin/streptomycin (Gibco, USA). The culture medium was changed every 2 days, and the hPDLCs of the 3rd to 5th generations were used for further experiments. Before the experiment, hPDLCs were characterized by immunofluorescence for the characteristic marker fibromodulin, as described previously (8).

Study participants were three volunteer laboratory members. As previously described (23), the density-gradient centrifugation method isolated human peripheral blood mononuclear cells (hPBMCs) from donated blood. We diluted 15 ml anticoagulant blood and 15 ml RPMI-1640 in a 1:1 ratio, and then 30 ml diluted blood was slowly added to the top of 15 ml lymphocyte separation solution (TBD, China). The PBMC layer was carefully extracted after centrifugation. Next, the cells were suspended in RPMI-1640 and centrifuged again. PBMCs were collected and resuspended in 1640 medium containing 1% FBS for cell sorting. Naive CD4^+^T cells from PBMCs were isolated using the Immunomagnetic Negative Selection Kit (STEMCELL, Canada) and seeded in 24-well plates using 1 × 10^6^ cells per well in 1 ml medium. Naive CD4^+^T cells were activated with 20 ng/ml IL-2 (PeproTech, USA) and 12 μL/ml CD3/CD28 (STEMCELL, Canada) for 3 days.

### Preparation of Conditioned Medium of hPDLCs Treated With rhIL-7 and Effect on CD4^+^T Cells

About 1 × 10^5^ hPDLCs were seeded in 6-well plates, and a fresh medium was introduced and supplemented with different concentrations of rhIL-7 (PeproTech, USA) after 24 h; cultivating was continued for another 24 h. Cells in the inhibitor (DAPT) group are pre-treated for 2 h at 37°C with 100 μM DAPT (MedChemExpress, USA) and then replaced with fresh medium supplemented with 200 ng/ml rhIL-7. The supernatants were then collected and used to incubate activated CD4^+^T cells for 3 days for subsequent experiments. CD4^+^T cells in the negative control group were cultured in an unconditioned medium, whereas cells in the control group were cultured in a conditioned medium of hPDLCs without rhIL-7-treated.

### Flow Cytometry Analysis

The supernatants of hPDLCs from each group were used to incubate with CD4^+^T cells for 3 days. The cells were collected, and a 2 μL/ml cell activation cocktail (with Brefeldin A) (BioLegend, USA) was added. Every 100 μl of this cell activation cocktail contains phorbol-12-myristate 13-acetate (40.5 μM), ionomycin (669.3 μM), and Brefeldin A (2.5 mg/mL) in dimethyl sulfoxide (DMSO) (×500). The cells were then cultured for 4–6 h and centrifuged to collect the activated cells. We added 300 μl of phosphate-buffered saline (PBS)-resuspended cells, 2 μl fluorescein isothiocyanate (FITC) anti-human CD4 (BioLegend, USA), 2 μl PE anti-human CD25 (BioLegend, USA), and 2 μl allophycocyanin (APC) anti-human CD127 (BioLegend, USA) for staining. After staining, the cells were incubated for 30 min. The cell precipitates were collected by centrifugation. The cells were resuspended in 100 μl fixation solution (BioLegend, USA), vortex oscillated for 30 s, and incubated for 30 min. Next, a 2-ml permeabilization buffer (BioLegend, USA) was added. The solution was centrifuged to collect the cell precipitates; 100 μl permeabilization buffer was added to resuspend the cells, and 2 μl Brilliant Violet 421 Anti-Human IL-17A (BioLegend, USA) was added for staining. The cells were then incubated for 60 min. The PBS solution was added to maintain a constant volume of 300 μL, and the ratio of CD4^+^ IL-17A^+^ Th17 cells and CD4^+^ CD25^+^ CD127^−^ Treg cells was determined by flow cytometry (FCM).

After digestion with 0.25% trypsin (Gibco, USA), the hPDLC precipitates in each group were collected by centrifugation. The cells were resuspended in 300 μl binding buffer after rinsing with PBS twice. The hPDLCs were stained with Annexin V-FITC/PI (KeyGEN Biotech, Nanjing, China) and incubated under dark conditions for 15 min. The apoptotic necrosis of hPDLCs was assessed by FCM.

The hPDLC precipitates from each group were collected by centrifugation after digestion with 0.25% trypsin (Gibco, USA); the cells were rinsed and resuspended in PBS. Next, 2 μl PE anti-human CD274 (BioLegend, USA) was added for staining and incubated for 30 min. The mean fluorescence intensity (MFI) of PD-L1 in hPDLCs was determined by FCM. Data were collected using FACSCalibur (BD LSRFortessa X20, USA), and the FlowJo software (San Carlos, CA. USA) was used for further analysis.

### Cell Counting Kit-8

Briefly, 1 × 10^4^ hPDLCs were seeded in 96-well plates for 24 h, a fresh medium was introduced and supplemented with rhIL-7 at different concentrations and cultured for 24 h. Next, CCK-8 was added (Dojindo, Japan) to each well and incubated at 37°C for 1 h in dark conditions. The absorbance was recorded at 450 nm.

### Enzyme-Linked Immunosorbent Assay

After hPDLCs were treated with different concentrations of rhIL-7 for 24 h, the supernatants of each well were collected, the concentration of TGF-β and IL-6 was determined using an ELISA Kit (Meimian, Wuhan, China), and optical density (OD) value was measured by an enzyme label instrument at a wavelength of 450 nm. Then, the sample OD values measured by different detection indexes were substituted into the corresponding standard curve regression equation to calculate the actual sample concentration.

After incubated in a conditioned medium of hPDLCs for 3 days, CD4^+^T cells were collected and lysed with radioimmunoprecipitation assay (RIPA) buffer containing 0.1 mM phenylmethylsulfonyl fluoride (PMSF) reagent and 2% phosphatase inhibitor cocktail (Beyotime, Shanghai, China). Homogenates were centrifuged at 14,000 × *g* for 10 min at 4°C, and the collected supernatants were used to detect the concentration of Foxp3 using an ELISA Kit (Meimian, Wuhan, China).

### Reverse Transcription Quantitative PCR

The hPDLCs were collected after being treated with different concentrations of rhIL-7 for 24 h. Following the instructions of the manufacturer, total RNA was isolated from hPDLCs using the TRIzol reagent (Thermo Fisher Scientific, USA). Total RNA was reverse-transcribed to complementary cDNA using Servicebio® RT First-Strand cDNA Synthesis Kit (Servicebio, Wuhan, China) according to the instructions of the manufacturer. Target genes were amplified in SYBR Green qPCR Master Mix (Servicebio, Wuhan, China) using ABI 7900 (Applied Biosystems, USA). Considering glyceraldehyde-3-phosphate dehydrogenase (GAPDH) as a reference gene, the relative gene expression of hypoxia-inducible factor (HIF)-1α, TCF7, Notch1, Jagged1, and Hes1 was quantified using the 2^−ΔΔCt^ method. Details of the primer sequences are listed in Table 1.

**Table 1:** Primers sequences of target gene.

**Gene**	**Forward (5^**′**^-3^**′**^)**	**Reverse (5^**′**^-3^**′**^)**
HIF-1α	GCTCATCAGTTGCCACTTCCAC	CCAAATCACCAGCATCCAGAAG
TCF7	GGTTCACCCACCCATCCTTG	TGCTTGTGTCTTCAGGTTGCG
Notch1	GGACGGCGTGAACACCTACAA	GCAGGCATTTGGCATCAGC
Jagged1	AGAGATGACTTCTTTGGACACTATGC	GCTCATTACAGATGCCGTGGA
Hes1	ATTCTGGAAATGACAGTGAAGCAC	CACCTCGGTATTAACGCCCTC

### Western Blot

The hPDLCs were rinsed with pre-cooled PBS and then lysed with the RIPA buffer containing 0.1 mM PMSF reagent and 2% phosphatase inhibitor cocktail (Beyotime, Shanghai, China). The hPDLCs were scraped off with a scraper after being lysed on ice for 15 min, and the supernatants were then collected after the homogenates were centrifuged at 4°C at 14,000 × *g* for 10 min. Protein concentrations were measured with an enhanced BCA Protein Assay Kit (Beyotime, Shanghai, China). Protein was mixed with 5× sample loading buffer (Beyotime, Shanghai, China) and boiled at 99°C for 5 min. Equal amounts of protein samples were separated by sodium dodecyl sulfate polyacrylamide gel electrophoresis (SDS-PAGE; Epizyme, Shanghai, China) for electrophoresis and then transferred to polyvinylidene fluoride (PVDF) membranes (Millipore, USA). The PageRuler™ Prestained Protein Ladder (10–180 kDa, Thermo Fisher Scientific, USA) was used to help locate and separate the target proteins. After blocking with 5% BSA (Meilunbio, Dalian, China) for 1 h, the membrane was incubated with primary antibodies of GAPDH (1:2,000, Proteintech, Wuhan, China), TGF-β1 (1:1,000, ABclonal, Wuhan, China), IL-6 (1:2,000, Proteintech, Wuhan, China), PD-L1 (1:2,000, Proteintech, Wuhan, China), Notch1 (1:600, Proteintech, Wuhan, China), Jagged1 (1:2,000, Proteintech, Wuhan, China), Hes1 (1:1,000, ABclonal, Wuhan, China), HIF-1α (1:2,000, Proteintech, Wuhan, China), and TCF7 (1:600, Proteintech, Wuhan, China) overnight at 4°C. The antibodies mentioned above were diluted with primary antibody dilution buffer (Servicebio, Wuhan, China). The membrane was incubated with an anti-rabbit or anti-mouse secondary antibody (1:5,000, Proteintech, Wuhan, China) diluted with secondary antibody dilution (Servicebio, Wuhan, China) for 1 h at room temperature after being washed with TBST. Then, protein bands were detected with ECL luminescence reagent (Meilunbio, Dalian, China). The protein level indicated by band intensity was quantitatively analyzed by the ImageJ software (National Institutes of Health, Bethesda, MD, USA).

### Statistical Analysis

Statistical analysis was performed using the IBM SPSS 20.0 (IBM Corp., Armonk, NY, USA) software. All data are shown as mean ± SD. Statistical significance was determined using Tamhane's T2 test, one-way ANOVA, or the Kruskal–Wallis test. A *p*-value < 0.05 was considered statistically significant.

## Results

### hPDLC Treatment With rhIL-7 Promoted the Polarization of CD4^+^T Cells Into Treg Cells

**Figure 1:**
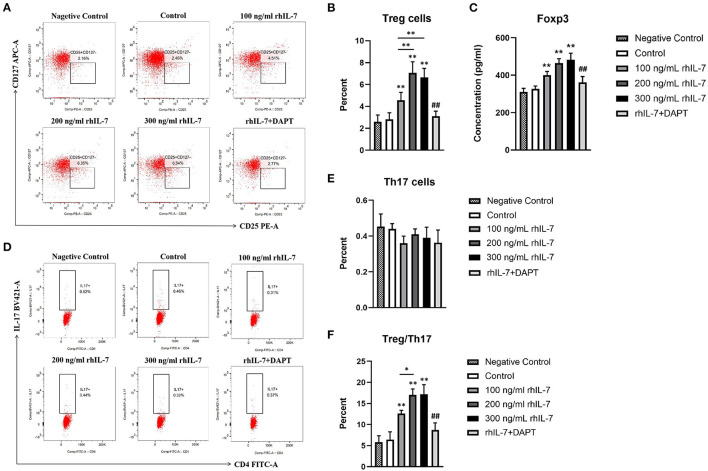
Effects of conditioned medium of human periodontal ligament cells (hPDLCs) treated with rhIL-7 on the differentiation of Treg/Th17 cells. The supernatants of hPDLCs were collected after being treated with different concentrations of rhIL-7 for 24 h and used to culture CD4^+^T cells for 3 days. Flow cytometry (FCM) was used to detect the polarization of CD4^+^T cells into Treg cells **(A,B)** and Th17 cells **(D,E)**. **(C)** The concentration of Foxp3 in CD4^+^T cells was detected by ELISA. **(F)** The ratio of Treg/Th17. Representative dot plots show the gating strategy of FCM analysis. *n* = 3, ^*^p < 0.05. ^**^p < 0.01. ^##^DAPT group compared with 200 ng/mL rhIL-7 group, *p* < 0.01.

We investigated the effect of a conditioned medium of hPDLCs treated with rhIL-7 on CD4^+^T-cell differentiation. The results showed that CD4^+^T cells cultured with supernatants of hPDLCs treated with rhIL-7 significantly upregulated the proportion of Treg cells. However, the proportion of Treg cells in the DAPT group was significantly reduced compared with the 200 ng/ml rhIL-7 group (Figures 1A,B). Although the proportion of Th17 cells decreased slightly, there was no statistical difference compared with the control group (Figures 1D,E). The concentration of Foxp3 in CD4^+^T cells and the ratio of Treg/Th17 showed the same trend as the proportion of Treg cells (Figures 1C,F).

### Effect of rhIL-7 on the Proliferation, Apoptosis, and Necrosis of hPDLCs

**Figure 2:**
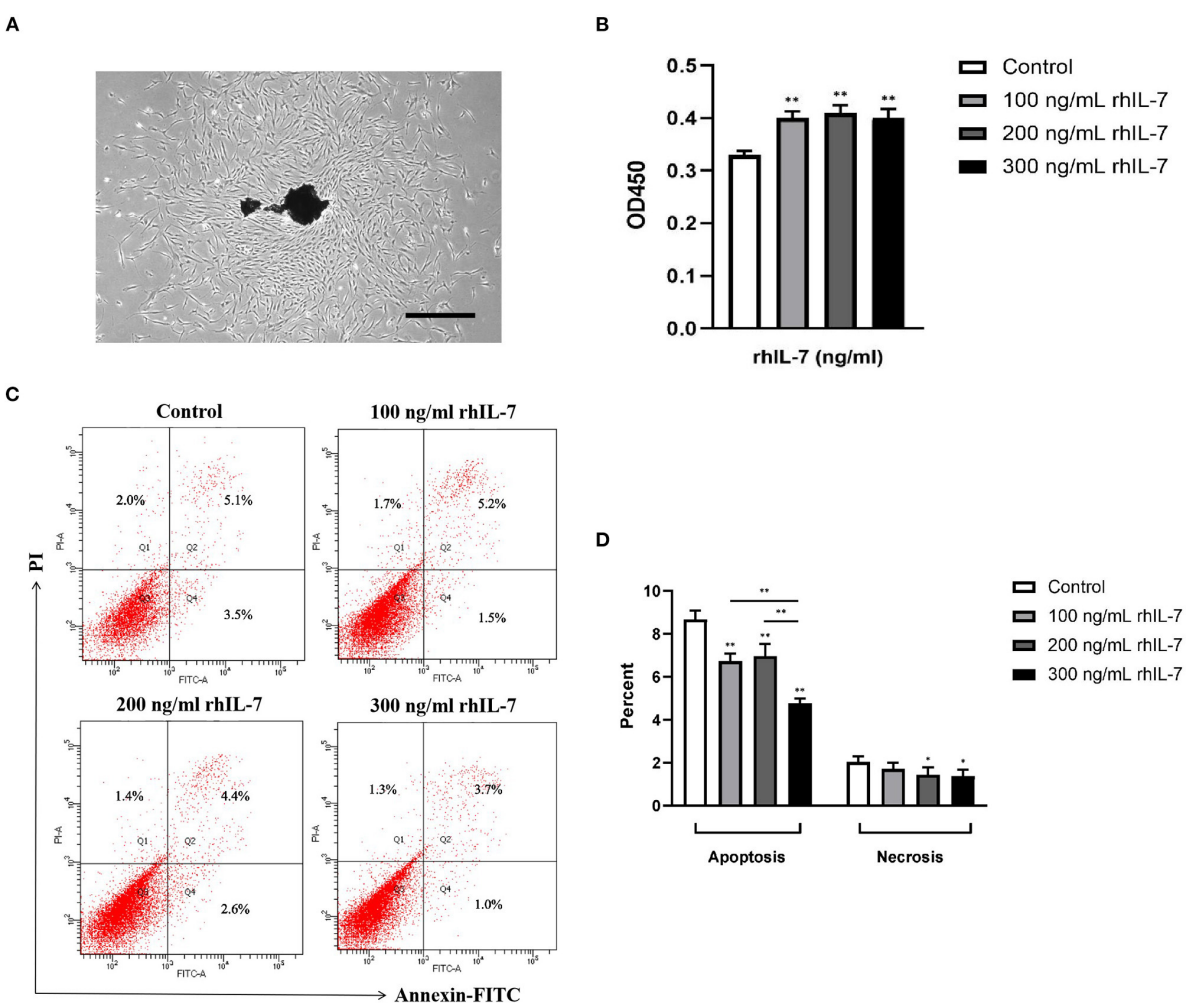
Primary human periodontal ligament cells. **(A)** Under the microscope, it can be seen that many fibroblast cells are crawling out around the dark brown primary tissue block. The cells are adherent to the wall and grow with a clear outline. Most of the cells are flat and spindle-shaped or star-shaped structures arranged in radial or whirlpool shape. Effect of rhIL-7 on the proliferation of hPDLCs. Scale bar = 1,000 μm. **(B)** After hPDLCs were treated with different concentrations of rhIL-7 for 24 h, CCK-8 was used to detect the cell survival rate of the hPDLCs. A summary of the results is shown in the bar graphs. Effect of rhIL-7 on the apoptosis and necrosis of hPDLCs. **(C,D)** We used Annexin V-FITC/PI staining to detect the proportion of apoptosis and necrosis in the cells of each group by FCM. Representative dot plots show the gating strategy of FCM analysis. The number in the scatter plot represents the percentage of cells in the corresponding state. *n* = 3, ^*^p < 0.05. ^**^p < 0.01. CCK-8, cell counting kit-8; FCM, flow cytometry; hPDLCs, human periodontal ligament cells.

We extracted primary hPDLCs for culture (Figure 2A). Our results showed that the presence of rhIL-7 at different concentrations significantly promoted the proliferation of hPDLCs (Figure 2B) and significantly reduced the proportion of apoptotic cells. The presence of rhIL-7 at higher concentrations also significantly downregulated the proportion of necrotic cells (Figures 2C,D), indicating that IL-7 could promote proliferation and inhibit the apoptotic necrosis of hPDLCs.

### Expression of Inflammatory Cytokines and Signal Molecules After RhIL-7 Treatment With HPDLCs

**Figure 3:**
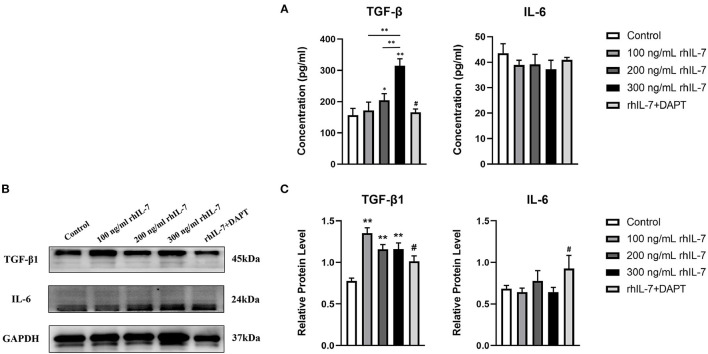
Effect of rhIL-7 on inflammatory factors related to the immune response in hPDLCs. **(A–C)** After hPDLCs were treated with different concentrations of rhIL-7 for 24 h, the concentration and protein level of TGF-β and IL-6 in the supernatants of each group were detected by ELISA and WB. *n* = 3, ^*^p < 0.05. ^**^p < 0.01. ^#^DAPT group compared with 200 ng/mL rhIL-7 group, *p* < 0.05. hPDLCs, human periodontal ligament cells; TGF, transforming growth factor; WB, Western blot.

**Figure 4:**
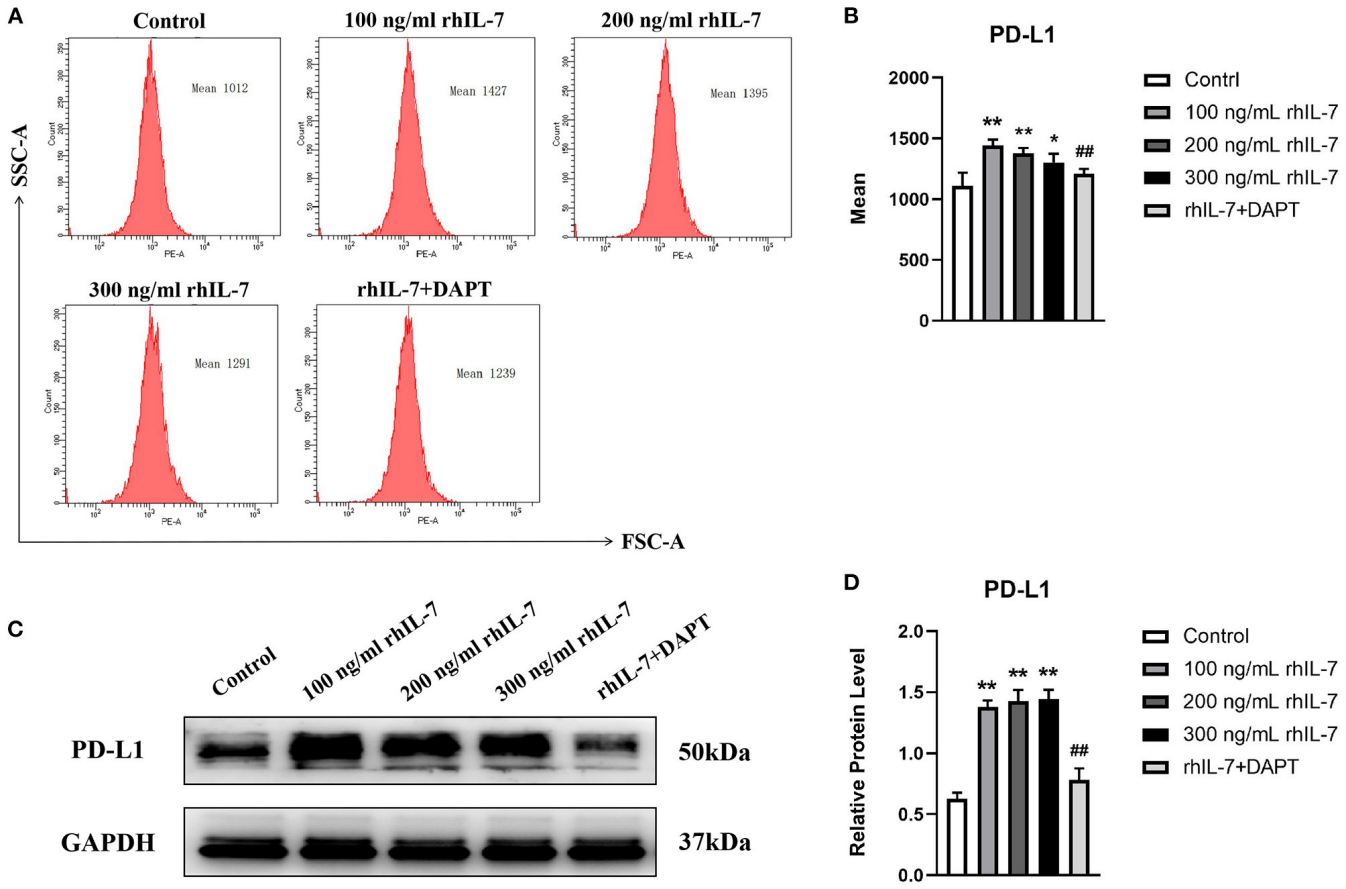
Effects of rhIL-7 on the expression PD-L1 in hPDLCs. **(A–D)** After hPDLCs were treated with different concentrations of rhIL-7 for 24 h, the MFI and the protein level of PD-L1 in each group were determined by FCM and WB. Representative histograms show the gating strategy of FCM analysis. *n* = 3, ^*^p < 0.05. ^**^p < 0.01. ^##^DAPT group compared with 200 ng/mL rhIL-7 group, *p* < 0.01. FCM, flow cytometry; hPDLCs, human periodontal ligament cells; MFI, mean fluorescence intensity; WB, Western blot.

**Figure 5:**
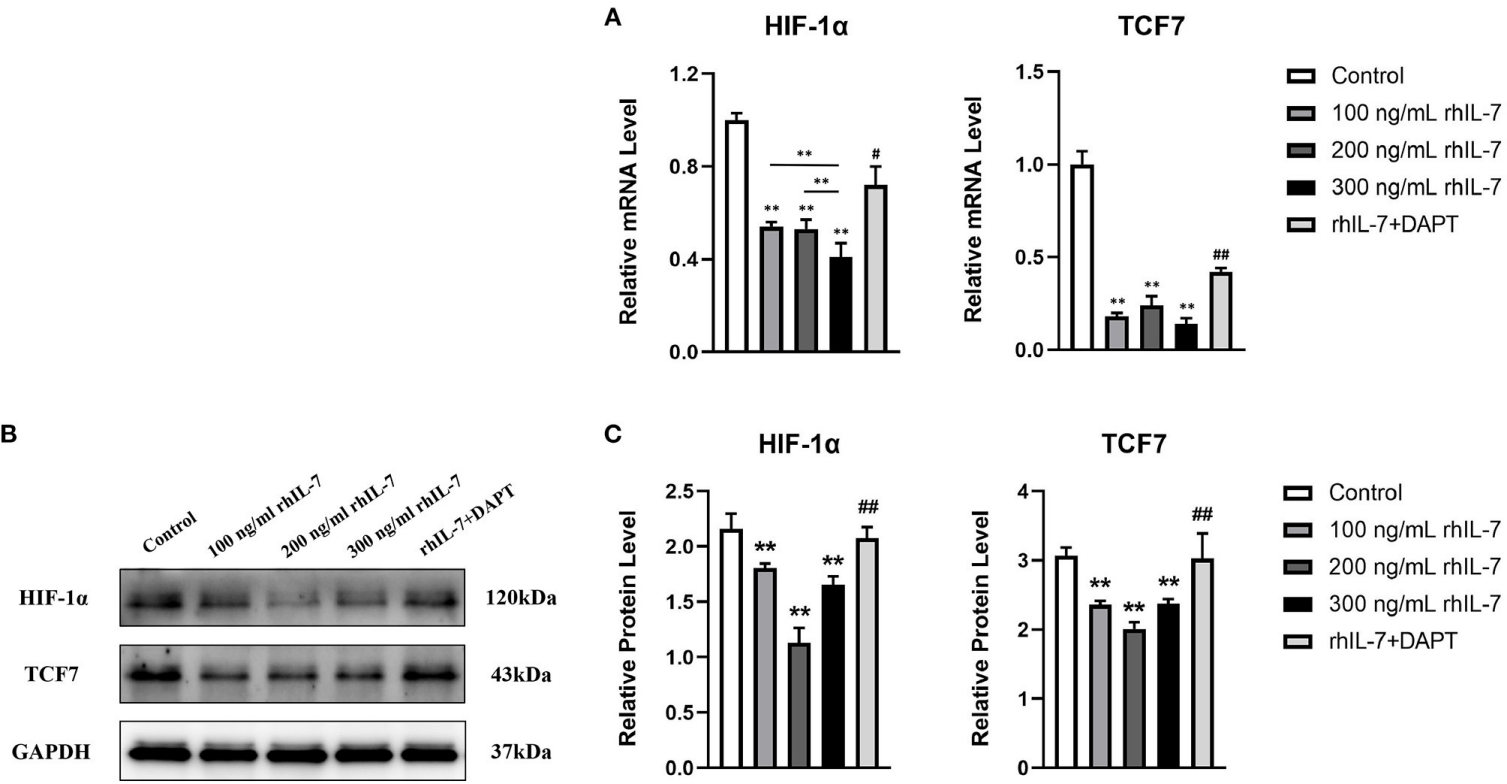
Effect of rhIL-7 on signal molecules related to the immune response in hPDLCs. **(A–C)** The mRNA and protein levels of HIF-1α and TCF7 in hPDLCs were detected by RT-qPCR and WB after treatment with different concentrations of IL-7 for 24 h. A summary of the results is shown in the bar graphs. *n* = 3. ^**^p < 0.01. ^#^DAPT group compared with 200 ng/mL rhIL-7 group, *p* < 0.05. ^##^DAPT group compared with 200 ng/mL rhIL-7 group, *p* < 0.01. HIF, hypoxia-inducible factor; hPDLCs, human periodontal ligament cells; RT-qPCR, reverse transcription quantitative PCR; WB, Western blot.

We speculated that IL-7 could affect the expression of immunomodulatory cytokines secreted by hPDLCs, thus further influencing the differentiation process of CD4^+^T cells. Therefore, we further studied the effects of rhIL-7 on hPDLCs and inflammatory factors and signaling molecules related to the immune response. The results of ELISA (Figure 3A) and WB (Figures 3B,C) showed that in the presence of rhIL-7, the concentration and protein level of TGF-β in the supernatants were significantly upregulated and significantly higher than that of IL-6. The addition of DAPT significantly inhibited the expression of TGF-β compared with the 200 ng/ml rhIL-7 group. The concentration and protein level of IL-6 decreased slightly but were already low, and no obvious change was noted. FCM (Figures 4A,B) and WB (Figures 4C,D) detection showed that in the presence of rhIL-7, the expression and protein level of PD-L1 on hPDLCs were significantly upregulated, whereas the DAPT group decreased significantly. RT-qPCR and WB analysis showed that in the presence of rhIL-7, the mRNA (Figure 5A) and protein levels (Figures 5B,C) of HIF-1α and TCF7 in hPDLCs were significantly decreased, whereas the addition of DAPT increased their expression.

### The Notch1 Signaling Pathway Is Activated After hPDLC Treatment With rhIL-7

**Figure 6:**
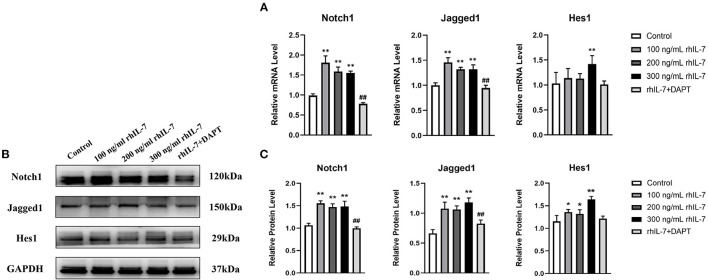
Effects of treatment with different concentrations of rhIL-7 in hPDLCs on Notch1 signaling pathway-related molecules. **(A–C)** After hPDLCs were treated with different concentrations of IL-7 for 24 h, the mRNA level and protein level of Notch1, Jagged1, and Hes1 were detected by RT-qPCR and WB. *n* = 3, ^*^p < 0.05. ^**^p < 0.01. ^##^DAPT group compared with 200 ng/mL rhIL-7 group, *p* < 0.01. hPDLCs, human periodontal ligament cells; RT-qPCR, reverse transcription quantitative PCR; WB, Western blot.

The results of RT-qPCR (Figure 6A) and WB (Figures 6B,C) demonstrated that in the presence of IL-7, the mRNA and protein levels of Notch1, Jagged1, and Hes1 in hPDLCs were increased, and the addition of DAPT inhibited their expression compared with the 200 ng/ml rhIL-7 group.

## Discussion

In this study, treatment of hPDLCs with IL-7 promoted cell proliferation and inhibited cell apoptosis, upregulated the expression of TGF-β and PD-L1 and inhibition of the expression of HIF-1α and TCF7 by activating the Notch1 signaling pathway, and promoted the polarization of CD4^+^T cells into Treg cells, thus regulating immune homeostasis in the periodontal microenvironment.

Interleukin-7 is an important cytokine of the immune system that plays a key role in maintaining T-cell homeostasis. IL-7 can promote the survival of Treg cells and maintain its immunosuppressive effect by upregulating the expression of Foxp3 (24, 25); nevertheless, this is contrary to previous research (17). IL-7 is also involved in periodontitis and orthodontic tooth movement by inhibiting the osteogenic differentiation of periodontal ligament stem cells through the mitogen-activated protein kinase pathway (26). CD4^+^T cells play a significant role in periodontitis and orthodontic tooth movement. Their function and differentiation can directly affect the local periodontal immune microenvironment and regulate the inflammatory response and osteogenic or osteoclastic activities. Local Treg/Th17 imbalance in periodontitis can promote inflammatory responses and can aggravate the absorption and destruction of alveolar bone (27). The present study showed that a conditioned medium of hPDLCs treated with rhIL-7 could induce Treg-cell differentiation, but had no significant effect on the differentiation of Th17 cells, indicating that IL-7 treatment could promote the immunosuppressive ability of PDLCs and participate in the maintenance of periodontal immune homeostasis by influencing T-cell differentiation.

Interleukin-7 plays an anti-apoptotic and pro-proliferative role by regulating the proteins of the Bcl-2 family to promote cell cycle progression and inhibit cell autophagy (28–30). The results of our study showed that rhIL-7 could promote the proliferation of hPDLCs and inhibit their apoptosis and necrosis. Our results are consistent with the biology of IL-7, which is consistent with the results of previous studies.

Both Th17 cells and Treg cells require the presence of a TGF-β signal in the initial differentiation stage. However, IL-6 can induce the polarization of Th17 cells by phosphorylation and activation of Stat3 and downregulate the expression of Foxp3 to inhibit the differentiation of Treg cells (31). TGF-β is a key regulatory factor of immune response and growth factor, which has a significant immunosuppressive effect, can promote the expression of Foxp3, promote the proliferation and differentiation of Treg cells, and maintain its immunosuppressive function (32). In periodontal tissues, TGF-β induces the expression of osteoblast differentiation proteins, such as ALP, and pro-regeneration factors, such as α-SMA (33, 34), by promoting PDLC proliferation, enhancing the synthesis of extracellular matrix protein of PDLCs and osteoblast differentiation to participate in alveolar bone remodeling and periodontal tissue remodeling, which are crucial processes for maintaining the immune homeostasis of the periodontal microenvironment and also tissue repair and regeneration (35, 36). In this study, IL-7 was found to significantly upregulate the expression of TGF-β in hPDLCs. In contrast, the IL-6 expression was low, and IL-7 had no significant effect on it, consistent with the differentiation results of CD4^+^T cells. Interestingly, in the absence of IL-7, the expression of TGF-β was significantly higher than that of IL-6, and the polarization ratio of Treg cells was significantly higher than that of Th17 cells, suggesting that PDLCs were involved in maintaining local immune homeostasis, and IL-7 enhanced the immunomodulatory effect of PDLCs.

Both HIF-1 and TCF7 promote inflammation by inhibiting Treg cells and inducing Th17 and Th1 cells. HIF-1α is a major transcription factor involved in hypoxia response, which can promote IL-17 expression and Th17 cell differentiation by inducing RORγt expression (37–39). HIF-1α also promotes PDLC apoptosis and inhibits osteoblastic differentiation in periodontal tissues (40–42). TCF7 plays a key role in the development and differentiation of T lymphocytes and inhibits the differentiation and maturation of Treg cells, whereas Foxp3 can inhibit the expression of TCF7 protein (43). The PD-1/PD-L1 signaling pathway is a negative feedback regulation mechanism of the immune response during the activation of T cells, in which PD-L1 is a negative costimulatory molecule (44). PD-L1 is involved in maintaining immune homeostasis by inhibiting the differentiation function of effector T cells, upregulating the expression of Foxp3, inducing differentiation of Treg cells, and enhancing their immunosuppressive ability (45). In this study, IL-7 significantly downregulated the mRNA expression levels of HIF-1α and TCF7 and significantly upregulated the expression level of PD-L1 in PDLCs. The results of our study are consistent with those of previous studies. These results suggested that IL-7 can participate in the immune regulation of periodontal microenvironment and maintain local immune homeostasis by influencing the expression of inflammatory factors and signal molecules in PDLCs.

The Notch signaling pathway is a highly conserved intercellular system that plays a decisive role in cell proliferation and differentiation (46). DAPT is a Notch-pathway-specific inhibitor. Downregulation of Notch1 and Jagged1 was accompanied by loss of bone, suggesting that the Notch1 signaling pathway plays a key regulatory role in periodontitis and bone metabolism balance (47). Activating the Notch signaling pathway in hPBMCs through Jagged1 can reduce cell senescence and cell cycle arrest, thus promoting tissue regeneration and repair (46). Furthermore, the Notch1 signaling pathway promotes the expression of TGF-β and the formation of Treg cells mediated by the upregulation of PD-L1 expression and phosphorylation of Smad3 (48–51). In addition, the Notch1 signaling pathway can enhance the proliferation, adhesion, and migration ability of PDLCs and can promote osteoblastic differentiation and angiogenesis by enhancing the expression of TGF-β, ALP, and α-SMA, thus participating in the tissue regeneration and repair (52–55). In this study, we found that IL-7 can promote the proliferation and inhibit the apoptosis of hPDLCs, upregulate the expression of PD-L1 and TGF-β, and promote the formation of Treg cells. Meanwhile, we observed a significant increase in the expression level of molecules related to the Notch1 signaling pathway. However, the addition of DAPT effectively reversed these effects. The results suggested that IL-7 may regulate the expression of immunomodulatory factors in PDLCs by activating the Notch1 signaling pathway, thus affecting the differentiation process of CD4^+^T cells.

In this experiment, we determined the effect of soluble mediators on Treg/Th17 cells polarization through conditioned medium application, which is the simplest method for cell interaction studies. The advantage of conditioned medium is that it can avoid the influence of target cells on conditioned cells, focus on the effect of conditioned cells on target cells, and allow observation of the activity of soluble factors (56, 57). As one of the methods of indirect co-culture, conditioned medium is very effective to observe the interaction between cells *in vitro*. However, compared to other indirect co-culture methods, such as transwell, conditioned medium has its limitations including nutrient deficiency, the uncontrollability of the optimal concentration and time distribution of these secretory factors, and the inability to study the chemotaxis of soluble factors on target cells (56). Therefore, it will be the focus of our next experiment to study the effect of IL-7-treated PDLCs on CD4^+^T cells through other co-culture methods.

In summary, we found that IL-7 acting on hPDLCs may regulate the expression of inflammatory factors and signaling molecules through the activation of the Notch1 signaling pathway, the expression changes of these immune regulatory factors further promote the differentiation of CD4^+^T cells into Treg cells, thus participating in the regulation of immune homeostasis in the periodontal microenvironment. TGF-β, as the endogenous cytokine of hPDLCs, seems to play a key role in regulating periodontal immune homeostasis through Notch signaling.

The self-renewing, multidirectional differentiating, and immunomodulatory characteristics of PDLCs are central to maintaining periodontal homeostasis. These properties and their high availability also reveal the potential usefulness of PDLCs as cell therapy in periodontal disease, regenerative medicine, autoimmune diseases, and inflammatory diseases (58, 59). Autologous periodontal membrane cell transplantation can be used to regenerate periodontal tissue defects (60–62). Our study suggests that PDLCs treated with IL-7 may have more immunosuppressive properties and enhanced ability of periodontal tissue regeneration and repair, which may contribute to the maintenance of periodontal tissue health in future clinical practice and the prevention and treatment of periodontal tissue defects caused by inflammation.

## Data Availability Statement

The raw data supporting the conclusions of this article will be made available by the authors, without undue reservation.

## Ethics Statement

The studies involving human participants were reviewed and approved by Ethical Committee of Southern Medical University. The patients/participants provided their written informed consent to participate in this study.

## Author Contributions

C-FL conceived and supervised the experiments. X-YY, C-FL, and Z-QZ wrote and edited the manuscript. X-YY, J-CH, and J-YL performed the experiments. X-YY and X-PC analyzed the data. All authors contributed to the article and approved the submitted version.

## Funding

This work was financially supported by the Natural Science Foundation of Guangdong Province, China (No. 2018A0303130261).

## Conflict of Interest

The authors declare that the research was conducted in the absence of any commercial or financial relationships that could be construed as a potential conflict of interest.

## Publisher's Note

All claims expressed in this article are solely those of the authors and do not necessarily represent those of their affiliated organizations, or those of the publisher, the editors and the reviewers. Any product that may be evaluated in this article, or claim that may be made by its manufacturer, is not guaranteed or endorsed by the publisher.
