# Supplementary material for: IL-7-Treated Periodontal Ligament Cells Regulate Local Immune Homeostasis by Modulating Treg/Th17 Cell Polarization
Source: Front Med (Lausanne). 2022 Feb 23;9:754341. doi: 10.3389/fmed.2022.754341 (PMC8905254; doi:10.3389/fmed.2022.754341)
Supplement: Supplementary file 1 [file Data_Sheet_1.docx]

https://www.jianguoyun.com/p/DS-etFcQrqDfCRix5YcE
